# Recent Development on the Electrochemical Detection of Selected Pesticides: A Focused Review

**DOI:** 10.3390/s20082221

**Published:** 2020-04-15

**Authors:** Jafar Safaa Noori, John Mortensen, Alemnew Geto

**Affiliations:** 1 IPM—Intelligent Pollutant Monitoring ApS, 2690 Karlslunde, Denmark; 2 Department of Science and Environment, Roskilde University, 4000 Roskilde, Denmark

**Keywords:** pesticides, glyphosate, bentazone, lindane, electrochemistry, sensors

## Abstract

Pesticides are heavily used in agriculture to protect crops from diseases, insects, and weeds. However, only a fraction of the used pesticides reaches the target and the rest slips through the soil, causing the contamination of ground- and surface water resources. Given the emerging interest in the on-site detection of analytes that can replace traditional chromatographic techniques, alternative methods for pesticide measuring have recently encountered remarkable attention. This review gives a focused overview of the literature related to the electrochemical detection of selected pesticides. Here, we focus on the electrochemical detection of three important pesticides; glyphosate, lindane and bentazone using a variety of electrochemical detection techniques, electrode materials, electrolyte media, and sample matrix. The review summarizes the different electrochemical studies and provides an overview of the analytical performances reported such as; the limits of detection and linearity range. This article highlights the advancements in pesticide detection of the selected pesticides using electrochemical methods and point towards the challenges and needed efforts to achieve electrochemical detection suitable for on-site applications.

## 1. Introduction

Water is one of the cheapest, yet the most valuable natural resource on the planet. Around 75% of the planet is covered with water and only 2% is fresh water that can be used by living organisms [1]. The freshwater percentage is subject to an ever-increasing pressure from heavy consumption, global warming, and most importantly contamination. Contamination is introduced to the water body by several sources, with agriculture being one of the main sources of pollution. Different chemicals are used in the agricultural industry to increase the crop yield, such as fertilizers and pesticides [2]. The extensive use of these chemicals has led to serious contamination issues in all the different water sources [3,4].

Applied fertilizers and pesticides can reach water sources either by surface run off or to groundwater by leaching through the soil. Heavy rain or extensive irrigation processes can further aggravate the leaching of contaminants to the groundwater [5]. Fertilizers and pesticides can cause serious health problems when constantly consumed by humans. Nitrate can cause blue baby syndrome and cancer [6] while some of the pesticides are classified to be carcinogenic [7]. Most of these chemicals are very persistent and do not degrade over time, but may adsorb to the soil particles and leach slowly to the groundwater causing continuous contamination [8,9,10,11]. Most chemical contamination cannot be removed by physical filtration, but requires sophisticated water cleaning which eventually will drive the water production cost to be higher than the price of the water, assuming that the contamination is ever discovered [3,4,12].

Due to the current available technology, contamination can take up to several years before it is discovered. Today, only snap shots of the real conditions are provided by the manual field sampling followed by the use of chromatographic methods, such as gas chromatography or high-performance liquid chromatography coupled with mass spectroscopy [6,13]. Even though chromatography is a very accurate, precise and selective technique, it is very expensive and time consuming and it only provides data for the moment the sampling took place. Water sources are very dynamic environments and contamination plumes can move from one location to another at relatively fast rates, especially in surface waters. This makes the current sampling techniques ineffective and may lead to potential hazardous water for the consumers. Continuous, automatic on-site measurements may be an answer to this problem and can provide a more thorough picture of the actual situation in the fields [14,15]. A technology that is growing to be a promising alternative suitable for such measurements is electrochemical sensing [14].

Electrochemical sensing depends on measuring an electrical quantity, such as current, charge or potential, as a result of the interaction between the targeted analyte and the sensing electrode. A typical electrochemical cell is composed of three electrodes: working electrode, counter electrode and a reference electrode. In voltammetric sensors, the reaction of interest usually occurs at the working electrode whose potential is monitored against the reference electrode and the resulting current is recorded between the counter electrode and the working electrode [16,17].

Electrochemical sensors were introduced in the 1960s by Clark and Lyons to measure glucose concentration in blood using oxygen sensitive electrodes [18]. These electrodes were then iterated and used in a variety of applications both for research and market purposes. The electrochemical sensor found its main application in medical industry and had wide benefits in diagnostic tools at the hospitals and as point-of-care devices [19]. One of the most famous applications of analytical electrochemistry is the glucose sensor which was introduced in the 1980s [20,21].

Even though electrochemistry has been applied in different fields, its application in the environmental sector is still limited. One of the electrochemical sensing applications common in the environmental analysis is the ion selective sensor. This technique has its own strength in terms of being able to selectively detect the targeted analyte [22]. However, the ion selective sensor needs frequent maintenance and calibration which drives the operation cost high and limits the concept of continuous measurements [23]. Even with this limitation, these sensors are still widely applied to conduct field testing in chemicals such as nitrate, fluoride and pH. Nevertheless, there are no actual sensors dedicated to measuring hazardous chemicals such as pesticides in water.

The environmental electrochemical sensing is an active subject where several studies have been focusing on developing sensors that can target pesticide substances directly in the field. Different methods have been tested and applied to achieve this goal such as immobilizing antibodies or enzymes that have certain affinity towards the targeted pesticide at the electrode surface to enable reaching low concentrations [24,25,26]. Molecular imprinted polymer (MIP) is one of the techniques that is also being heavily explored. The technique uses the principle of creating a negative imprint of the targeted molecule on the electrode surface to achieve better selectivity and sensitivity [27,28]. Another approach that has recently been gaining attention is to immobilize microorganisms on the electrode surfaces and monitor the interaction between these organisms when they get in contact with the targeted analyte [29,30]. An alternative method is to modify the electrode surfaces with nanomaterials such as carbon nanotubes or manganese dioxide nanotubes which will result in increased surface area, increased conductivity and hence better response [31,32]. In other cases, applying nanomaterials with the opposite charge of the targeted analyte will result in attracting the molecule towards the electrode surface, which will lead to enhanced performance [31].

**Figure 1:**
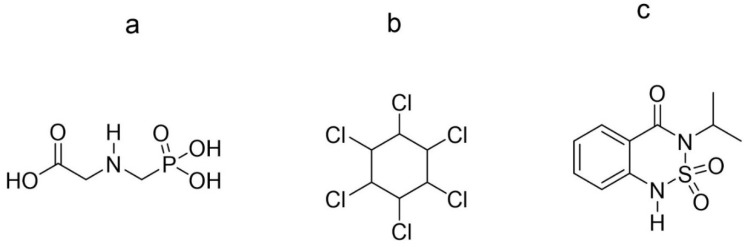
Chemical structure of (**a**) glyphosate, (**b**) lindane (**c**) bentazone.

In this report, the recent development in the electrochemical detection of three selected pesticides, i.e., glyphosate, lindane, and bentazone, is reviewed. Lindane is banned from use in agriculture by international conventions due to its high toxicity [33], whereas glyphosate and bentazone are two of the most widely used pesticides frequently detected in ground water and surface water beyond the regulatory limits set by the Environmental Protection Agency (EPA) [34]. The regulatory limit of pesticides in drinking water is 0.10 µg/L which corresponds to 0.59 nM, 0.34 nM and 0.42 nM concentration for glyphosate, lindane, and bentazone, respectively. The chemical structures of the three selected pesticides are shown in Figure 1.

The focus of this review is limited to chemical and biosensors for analytical purpose and does not include spectrometric, chromatographic, and chemiluminescence methods of analysis. Moreover, electrochemical and photocatalytic redox reports aimed at environmental remediation and cleaning purposes are also beyond the scope of this review.

## 2. Electrochemical Detection of Glyphosate

Glyphosate is one of the most commonly used pesticides in the world under the trade name Roundup. Glyphosate prevents plants from producing proteins necessary for growth and in this way kills weeds and grasses in competition with crops. Glyphosate and its degradation products are associated with development of resistant weeds and microorganisms, in addition to causing toxic effects in living organisms [35,36].

Glyphosate has been reported to be detected using bare and modified electrodes made of gold [28,37,38,39,40,41,42,43,44], copper [45,46,47], carbon [48,49,50,51,52,53,54], mercury [55,56,57], and platinum [58,59]. Electrochemical methods, type of electrodes, analytical performance, measurement condition, and sample matrix for glyphosate determination are summarized in Table 1.

The main challenge in the electrochemical detection of glyphosate is its poor electroactivity under accessible potential window using conventional electrodes and media [51,54]. Thus, indirect ways of detection were reported after converting into *N*-nitroso derivative which can easily be reduced and determined at mercury electrodes [55,56]. The secondary amino group in glyphosate molecule can easily be converted into *N*-nitroso group when treated with nitrous acid [57]. Using this approach, it was possible to determine glyphosate at a detection limit of 0.20 µM in water [55] and 0.08 µM in soil, water, and vegetables after sample preparation and derivatization in a column [56].

Molecularly imprinted polymer (MIP) is an attractive approach in electrochemistry since it allows to specifically recognize target molecules in preference to other closely related compounds based on shape, size, and functional groups [38]. As a result, gold electrodes modified with a molecularly imprinted polymer were developed for glyphosate detection [28]. The electropolymerisation of *p*-aminothiophenol-functionalised gold nanoparticles was conducted in the presence of glyphosate template molecules. After extracting the template, cavities with a similar shape to glyphosate were formed and allowed for the specific recognition of glyphosate that would bind to aniline moieties. The detection of the bound glyphosate molecules was achieved by linear sweep voltammetry (LSV), resulting in a linear range of 5.91 nM to 5.91 µM and a limit of quantification of 4.73 nM.

A pencil graphite electrode was also modified with gold nanoparticles and doubly imprinted nanofilm for the simultaneous determination of glyphosate and glufosinate using *N*-nitroso glyphosate and glufosinate as template molecules [52]. This approach is reported to separate overlapping reduction peaks for glyphosate and glufosinate by 265 mV enabling a selective determination for the pesticides. The detection limit of the sensor was 2.0 nM and 1.0 nM for glyphosate and glufosinate, respectively, in a linear range of 0.024–1.04 µM for glyphosate.

**Figure 2:**
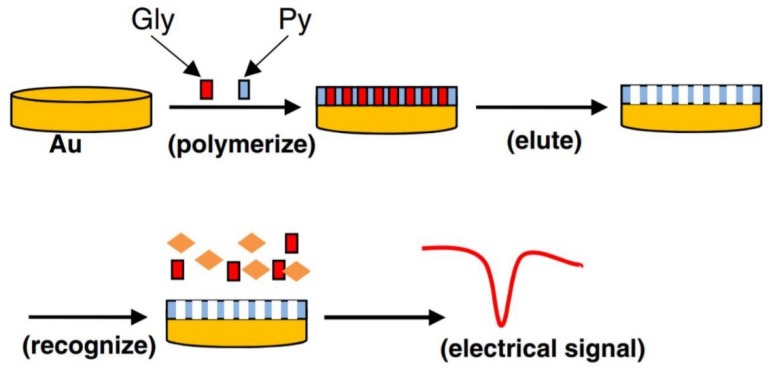
Molecularly imprinting for the electrochemical detection of glyphosate [38].

Zhang et al. [38] also developed a glyphosate moleculary imprinted polypyrrole-modified gold electrode (MIPPy) by electropolymerizing glyphosate and pyrrole using cyclic voltammetry. After polymerization, the embedded glyphosate molecule was extracted from the polypyrrole membrane with an overoxidation method (see Figure 2). The imprinted modified electrode was then successfully applied to determine glyphosate in cucumber and water samples using differential pulse voltammetry (DPV) in 0.10 KCl solution. The sensor response was linear between 0.03 to 4.73 µM and the calculated detection limit was 1.60 nM.

Carbon is often used as a low-cost, versatile and easy-to-handle material for electrochemical sensing. Several articles report the use of carbon as the working electrode for glyphosate detection [48,49,50,51,52,53,54]. Oliveira et al. [49] used a simple carbon paste electrode (CPE) for the oxidation of the isopropylamine salt form of glyphosate. Cyclic voltammetry (CV) at the electrode in the presence of glyphosate in buffer solution of pH 5 showed a clear oxidation peak at 0.95 V (vs Ag/AgCl). Under the optimum conditions, it was possible to determine the pesticide in milk, orange juice and agricultural formulation with a detection limit of 2.0 nM.

A pencil graphite-based electrode (PGE) modified with multi-walled carbon nanotubes-ionic liquid (MWCNTs-IL) and copper oxide (CuO) nanoparticle composite was also utilized for glyphosate sensing in soil and river water samples. The excellent electrical property of CuO and its complex forming property with glyphosate coupled to the electrical conductivity and high surface area of MWCNTs-IL attributed to the improved sensitivity and effectiveness of the electrode [53].

Similarly, a glassy carbon electrode modified with copper phthalocyanine/multi-walled carbon nanotube(GCE/MWCNT/CuPc) film has been used for the determination of glyphosate by electrochemical oxidation using DPV [48]. The authors reported that the strong interaction between glyphosate and copper ions to form a stable complex contributed the indirect detection of glyphosate at −50.0 mV vs. SCE based on Cu(I)/Cu(II) couple. Using this method, glyphosate was determined in the concentration range of 0.83–9.90 µM, with a detection limit of 12.20 nM.

High performance liquid chromatographic method was coupled to a gold electrochemical detector for the determination of glyphosate in environmental samples [39] and in urine and serum samples [62]. The detection principle is based on the current generated when carbohydrates, amino acids, amines, and sulfur compounds adsorb on gold and platinum electrodes under alkaline conditions. The current for glyphosate is reported to result from the adsorption through the non-bonded lone pair of *N*-atom on the oxide free gold surface of the electrode where it immediately oxidizes and adsorbed under alkaline condition. This method was successfully applied for the determination of glyphosate at the detection limit of 1.89 µM [39] and 0.30 µM [62].

However, a recent study [37] showed the possibility of glyphosate determination amperometrically at screen printed gold electrode without the need to any surface modification, sample preparation, chromatographic separation or pH adjustment. In this report, glyphosate showed an oxidation peak around 0.78 V in a normal water medium and it was possible to establish a linear curve between 2.0–300 µM with a detection limit of 1.60 µM. Even though the limit of detection is not low enough to meet legal requirements, this report is an interesting attempt to develop a functional sensor towards real application.

Electrochemical methods based on biosensors have also been described for glyphosate determination [40,44,51,60]. One such sensor was constructed by immobilizing the enzyme horseradish peroxidase (HRP) electrostatically onto a rotating gold disk electrode modified with poly(2,5-dimethoxyaniline)-poly(4-styrenesulfonic acid) (PDMA-PSS) nanoparticles. This biosensor was successfully used for glyphosate analysis on spiked corn samples within a concentration range of 0.012–0.46 µM and a detection limit of 0.59 nM [44]. In a similar principle, a horseradish peroxidase-based biosensor was investigated without the rotating disk arrangement. Measurements were conducted in a cell containing phosphate buffer solution and 0.70 M H_2_O_2_. The measuring principle was dependent on the principle of glyphosate inhibiting the signal obtained from H_2_O_2_. The detection limit was reported to be 0.01 µM [40].

An electrochemical immunoassay with antibody-modified magnetic particles was used to provide selective detection of glyphosate. Anti-glyphosate-IgG modified magnetic beads (MBs) and HRP-conjugated-glyphosate (tracer) were used in the immunoassay and the current was monitored as a function of the reduction of the enzymatic product tetramethylbenzidine in the presence of glyphosate. This way, it was possible to obtain a calibration curve in the range between 0.29 nM and 5.90 nM [60].

In another study [51] graphite-epoxy electrode modified by depositing multi-walled carbon nanotubes and horseradish peroxide (GE/MWCNTs-HRP) using electrophoresis was reported for measuring glyphosate. The measurement with this sensor was conducted in an electrolyte solution and depended on inhibition of the H_2_O_2_ reduction at the electrodes in the presence of glyphosate. The detection limit of this sensor was reported to be 1.32 pM.

## 3. Electrochemical Detection of Lindane

Lindane is an organochlorine insecticide used against insects that compromise fruit and vegetables. It is also used in formulations of lotions and shampoos to treat lice and scabies [64]. The mechanism of action of lindane is that it gets absorbed in the exoskeleton of parasites and leads to paralysis and death [65]. Exposure to lindane has been shown to elicit immunotoxic, reproductive, and developmental problems in laboratory animals, aquatic organisms, and humans [65,66,67]. The degradation products of lindane are also toxic, even though it is not easily degradable and is known to have a strong persistence in the environment. As a result, it tends to bioaccumulate and has been detected in human blood, breast milk, and adipose tissue from samples taken around the world [67]. Its well-established neurotoxicity, carcinogenicity, and consequent health risks led to a worldwide ban of lindane by the Stockholm Convention in 2007 [68,69]. In fact, some countries are still using it for economic reasons [30,64]. Further, it is still found in ecological niches such as water bodies and in crops, resulting in major environmental problems [68].

Electrochemical methods of analysis are not widely available for lindane due to its poor aqueous solubility and high negative reduction potential. However, some attempts have been reported to electrochemically reduce it in a completely organic [70,71,72,73,74] or mixtures of aqueous-organic [32,64,67,75,76,77] media. Various electrode materials were employed for the reduction including bare and modified carbon [32,64,70,71,73,75,76,77], silver [67], platinum [74], and copper [27].

A polarographic study of lindane at a mercury coated platinum electrode in dimethyl sulfoxide showed only a single reduction peak at −1.50 V vs. SCE and it has been concluded that the reduction takes place through a six-electron transfer process to form benzene. Though no other intermediate chlorinated compounds were detected in either type of reduction [74]. However, it has been known that certain metals, such as cobalt can catalyze a stepwise dechlorination (i.e., progressive chlorine loss) of lindane and other organohalides [78].

A direct reduction of lindane at a glassy carbon cathode in dimethylformamide (DMF) containing tetra-n-butylammonium tetrafluoroborate (TBABF_4_) exhibited two cathodic peaks at −1.40 V and −2.10 V. The first cathodic peak being attributed to the reduction of lindane itself, whereas the second peak is due to the reduction of chlorobenzene that is derived from lindane. This revealed that lindane undergoes a six-electron reduction process that affords benzene as a major product along with small amounts of chlorobenzene [67,71].

Another report investigated the reduction of lindane at silver electrodes in various reaction media [67,69]. A combination of one- and two-electron processes has been proposed to account for benzene as the major product. Dechlorination is essentially complete in DMF and in mixtures of water with DMF, CH_3_CN, and ethanol, whereas some chlorobenzene was detected in pure ethanol and CH_3_CN.

Kumaravel et al. [64] developed electroanalytical sensor using cellulose acetate modified glassy carbon electrode(CA/GCE) and used for the direct reduction of lindane in aqueous-alcoholic medium. The reduction potential of lindane on this modified electrode appeared at −1.50 V. The peak current at the electrode was linear from 50 to 180 µM with a detection limit of 9.18 µM. The analytical utility of the proposed method was evaluated by analyzing commercial lindane lotion and drinking water samples.

Fayemi et al. [76] also evaluated sensors based on PANI/Zn, Fe(III), and Nylon 6,6/MWCNT/Zn, and Fe(III) oxides nanofibers for the electrochemical reduction of lindane. The dynamic range for the lindane determination was demonstrated between 9.90 pM and 5.0 µM with the lowest detection limit of 32.0 nM obtained at Nylon 6,6/MWCNT/Fe_3_O_4_.

A non-enzymatic detection method of lindane by using CuO–MnO_2_ hierarchical nano-microstructures modified electrode was developed [32]. Cyclic voltammetric responses at the electrode was followed before and after the addition of lindane which the presence of lindane showed a distinct peak at −1.5 V (vs Ag/AgCl) in 60:40 methanol–water containing 0.05 M TBAB. At optimum conditions, the method enabled the detection of lindane in the concentration range of 1.0–700 µM at a detection limit of 4.80 nM.

Similarly, the catalytic effect of α-MnO_2_ nanostructures for the reduction of lindane was investigated. At the modified electrode a good linearity was established in the range of 1.10 to 510 µM with a detection limit of 114 nM. The proposed lindane sensor was also successfully employed for the determination of the pesticide in tap water samples [75]. In another study, NiCo_2_O_4_ was used as electrode modifier and employed for the detection of lindane in 0.05 M TBAB solution in 60:40 (v/v) methanol–water medium. The practical utility of the method was evaluated by analyzing spiked tap water sample in aqueous-alcohol mixture. The modified electrode exhibited the sensing abilities in the concentration range of 10.0–170 µM with a lower detection limit of 3.60 µM [77].

**Figure 3:**
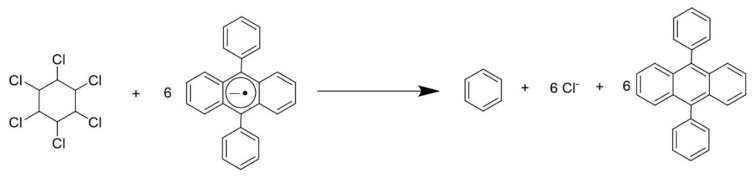
Proposed mechanism of 9,10-diphenylanthracene (DPA) mediated electrochemical reduction of Lindane [70].

An indirect reduction of lindane at vitreous carbon disk electrode was reported using 9,10 diphenylanthracene (DPA) as electrochemical mediator. In DMF solution, DPA showed an irreversible reduction peak at −1.79 V (vs Ag/AgCl) that shifted by 30.0 mV and a significant increase in peak current in the presence of lindane. The proposed mechanism (Figure 3) of mediated reduction is believed to occur by the rapid reaction of the DPA anion radical with lindane and the initial electron transfer is followed by further reduction so that the lindane undergoes an overall 6-electron (6e-) reduction by reaction of the DPA anion radical with intermediates [70]. Under optimum conditions, it was possible to determine lindane in the range 40.0–1000 µM.

A potentiometric approach was also demonstrated at a multi-walled carbon nanotubes modified copper electrodes [27]. After grafting the nanotubes to the electrode, the surface was further modified by molecular imprinting of lindane as the template. The developed method enabled the detection of lindane at a lowest limit of 1.0 nM. In general, most of the lindane detection methods have employed voltammetric techniques, however, one study reported lindane detection using electrochemical impedance spectroscopy (EIS) as the transduction method. The biosensor itself was based on the activity of Steptomyces strain M7, which utilizes lindane as the carbon source for growth. The biosensor reached a detection limit of 0.03 µM [30]. Table 2 summarizes electrochemical methods and main performance characters reported for the determination of lindane at different electrodes.

## 4. Electrochemical Detection of Bentazone

Bentazone is a selective pesticide acting as a photosynthetic electron transfer inhibitor to control sedges and broad-leaf weeds in corn fields, rice paddies, and other intensive crops [79,80]. Thus, selective crops like beans, maize, pepper and rice can survive the exposure to the pesticide [81,82]. Deposition from aerial applications, leaching, or run-off from agricultural lands [83] together with its low soil sorption and high water solubility led to bentazone contamination of ground- and surface water [80]. Hence, it has been reported as one of the most important contaminants in terms of frequency of detection and maximum concentration in surface freshwater and groundwater [79]. For example, in Denmark, it was one of the pesticides detected in 49.5% of the groundwater monitoring wells in the period 1990–2015 [84].

Ingestion of higher doses of bentazone through, e.g., drinking water may lead to acute toxic symptoms like vomiting, irregular breathing and irritation of the skin and eyes. However, repeated exposure to bentazone may lead to effects on reproductivity, mutagenicity, carcinogenicity, and organ toxicity [85].

Electroanalytical techniques have been used for the determination of bentazone at different electrodes with or without surface modification [31,86,87,88,89,90,91,92,93]. Overview of the electrochemical methods of analyses for bentazone determination is summarized in the Table 3. The use of a bare glassy carbon electrode for the determination of bentazone in commercial herbicides has been reported [86]. Using square wave voltammetry (SWV), a linear calibration plot was obtained between 15.0 and 22.60 µM of bentazone with a detection limit of 10.0 µM. The mechanism of bentazone oxidation is proposed to proceed on the nitrogen atom of the tertiary amine and corresponds to a one-electron transfer followed by slow chemical dimerization step of the resulting products [86,89], which also get adsorbed and lead to severe electrode surface fouling. This strong adsorption of redox products means that reusing the sensors is not an option, which eliminates the possibility of continuous on-site use. This issue has been suggested to be overcome by adding triton surfactant to the sample to avoid some of the adsorption [86].

Similarly, an amperometric detection coupled to a flow injection analysis(FIA) system at a glassy carbon electrode has been proposed by Cerejeira et al. [93]. Analysis of bentazone in estuarine waters was performed by means of calibration curves over the concentration range 2.50 to 50.0 µM at an oxidation potential of 1.10 V in acetate buffer solution (ABS) of pH 4.5.

In another report, a glassy carbon electrode modified with a film of polyaniline-carbon nanotubes-cyclodextrin (PANI-β-CD-MWCNT) was used for the detection of bentazone [31]. The electrode was employed for the direct oxidative determination of bentazone in pure and natural water samples by cyclic voltammetry in the range of 10.0–80.0 µM, with a detection limit of 1.60 µM.

A study conducted by Jevtic et al. [89] presented a way to measure bentazone in water using unmodified boron-doped diamond (BDD) using DPV. The measurements were conducted in the presence of a supporting electrolyte, Britton–Robinson (B–R), and a modified pH sample of 4. The limit of detection using this method was reported to be 0.50 µM.

We recently reported electrochemical determination of bentazone using a simple home-made screen-printed carbon electrode (SPE) and successfully applied to quantify bentazone in spiked ground and lake water samples. A simple low-cost fabrication process was followed to produce the electrodes which can be used repeatedly without significant activity loss. Square wave voltammetric (SWV) method was used to plot the calibration curve and quantify bentazone based on its oxidation without the need for electrode modification. The calibration plot was linear in the range 0.19–50.0 µM with a detection limit of 34.0 nM [90].

## 5. Future Perspective

The security trend, internet of things and market trends present a pressing need for a technology that can be reliable, fast and accurate in providing data on demand and alert in case of an incident. Thus, *in-situ* monitoring and point-of-care testing is highly needed in biomedical, pharmaceutical, industrial, and environmental analyses, where no pre-treatment before use or cleaning between measurements is required [94].

Electrochemical measuring technology appear to promise a strong option that can meet the market demand. However, the theoretical background of analytical electrochemistry strongly recommends introducing a supporting electrolyte to the sample of interest. Such a requirement for sample pre-treatment is considered one of the limiting factors for moving this technology to the field application level. However, environmental samples such as surface and groundwater, the amount of natural salts and nutrients could play an important role of being a replacement for artificial electrolytes and sample treatment. The other argument against utilizing electrochemistry for contamination detection will be the interference from other compounds that co-exist in the site. Using biologically functionalized electrodes in the fields may be the appropriate choice in this matter, but it may pose a risk of introducing more contamination to the environment in addition to the inherent poor stability of biomolecules. This may be overcome by maturing the molecular imprinted techniques, which is a strong candidate that can function without biological modification and has the potential to achieve the desired selectivity. Moreover, the development of material science and nanotechnology will offer a broad choice of novel materials for electrode fabrication or modification to meet the current challenges in the real continuous monitoring of pollutants.

## Figures and Tables

**Table 1:** Overview of reports on electrochemical methods, measurement conditions, analytical performance and sample matrix for the determination of glyphosate.

Electrode	Technique	Medium	pH	Potential	LOD	Linear Range	Matrix	Reference
Anti-glyphosate-IgG magnetic beads	Amperometry	0.10 M Citrate/PBS	5	−0.1 V vs. Ag/AgCl	0.03 nM	0.29 nM–5.90 nM	Beer sample	[60]
HRP/PDMA-PSS/Au	Amperometry	PBS		−0.1 V vs. Ag/AgCl	0.59 nM	0.01–0.46 µM	Spiked corn sample	[44]
HRP/PDMA-PSS/Au	Amperometry	0.10 M PBS	6.1	−0.28 V vs. Ag/AgCl	0.95 nM	0.01–0.47 µM		[43]
SPE/Chi/CNO/TYR	Amperometry	20.0 mM PBS	7	−0.2 V vs. Ag/AgCl	6.50 nM	0.02–10.0 µM	Water and soil	[61]
HRP/PDMA-PSS/Au	Amperometry	PBS	6.1	−0.28 V vs. Ag/AgCl	10.0 nM	1.50 nM–0.082 µM		[40]
Porous copper nanowires	Amperometry	0.10 M PBS in 0.10 M KCl	6.5		10.0 nM	0.01–5.0 µM	Fresh Fruit, Vegetables	[46]
Au	Amperometry	0.10 M NaOH			0.30 µM	0.59–268 µM	urine, serum	[62]
NiAl-LDH/Pt	Amperometry	0.10 M NaOH	12.8	0.49 V vs. SCE	1.0 µM	0.01–0.90 mM		[59]
Au	Amperometry	0.10 M NaOH	13	1.0 mV vs. SHE	1.89 µM	5.9 µM–1.06 mM	Extracted river water	[39]
Gold SPE	Amperometry	Tap water		0.78 V	2.0 µM	18–300 µM	Ground water	[37]
GCE/MWCNTs-HRP	CV	wide range buffer	4	−0.40 V vs. SCE	1.32 pM	0.10 nM–11.0 µM	Maize kernels	[51]
Cu/CPE, Cu/GCE	CV	0.10 M PBS	6.5			0–0.59 mM		[54]
Cu	Coulometry	0.03 M PBS/Methanol	6.8	0.05 V vs.	0.59 µM	0.59–200.0 µM	Tomato juice	[47]
MIP/GNPs-PGE	DPASV	ABS	5.5	−0.90 V vs. Ag/AgCl	2.0 nM	0.024–1.04 µM	Soil and human serum	[52]
HMDE	DPP	1.0 HCl		−0.70 V vs. Ag/AgCl	0.08 µM	0.06–10.4 and 23.6–591.5 µM	Water, soil, vegetable	[56]
Dropping Mercury Electrode	DPP	0.10 M HCl		−0.80 V vs. SCE	0.20 µM	0.20–1.24 µM	Tap water	[55]
Cu-BTC MOF/ITO	DPV	0.10 M PBS	5.5	0.10 V vs. SCE	0.14 pM	1.0 pM–10.0 µM	Green vegetable	[45]
HF-PGE/CuO/MWCNTs–IL	DPV	0.10 PBS	7	0.65 V vs. Ag/AgCl	1.30 nM	5.0 nM–1.10 µM	Soil and river water sample	[53]
MIPPy/Au	DPV	0.10 M KCl		0.20 V vs. SCE	1.60 nM	0.03–4.73 µM	Cucumber, Tap Water	[38]
GCE/MWCNT/CuPc	DPV	0.10 M PBS	7.4	−0.10 V vs. SCE	12.20 nM	0.83–9.90 µM		[48]
Cu^2+^-Cu/GCE	DPV	ABS	6	−0.015 V vs. Ag/AgCl	0.19 µM	5.0–60.0 µM	Drinking water	[50]
Electro-aggregated silver carbonate modified-Pt	DPV and LSV	0.1 M Na_2_CO_3_			40.0 µM	0–3.80 mM		[58]
MIP-MOF	LSV	10.0 mM [Fe(CN)6]^3–/4–^	7.2	−0.05 V vs. SCE	4.73 nM	5.91 nM–5.91 µM	Tap water sample	[28]
PPY-MIP/Au and PPy-MIP/ZnO	SWV	LiClO_4_		0.50 V vs. SCE	0.10 pM	0.10 pM–100 µM		[41]
PPY-MIP/Au	SWV	0.01 M LiClO_4_	5	0.38 V vs. SCE	1.0 pM	0.10 pM–10.0 µM		[42]
HMDE	SWV	1.25 M HCl		−0.70 V vs. Ag/AgCl	0.15 nM	0.30 nM–0.59 µM		[57]
CPE	SWV	0.20 M BR buffer	5	0.95 V vs. Ag/AgCl	2.0 nM	0.04–2.80 µM	Milk, orange juice, agricultural formulation	[49]
Atemoya peroxidase immobilised on modified nanoclay	SWV	0.10 M PBS	7	−0.10 V vs. Ag/AgCl	0.18 µM	0.59–26.90 µM	Spiked water	[63]

Abbreviations: **ABS**—acetate buffer solution; **CPE**-carbon paste electrode; **Cu-BTC MOF/ITO**—Cu-benzene-1,3,5-tricarboxylic acid-metal organic frameworks/Indium thin oxide; **Cu/CPE**—Cu/carbon paste electrode; **Cu/GCE**-Cu/glassy carbon electrode; **CV**—cyclic voltammetry; **DPASV**—differential pulse anodic stripping voltammetry; **DPP**—differential pulse polarography; **GCE/MWCNTs-HRP**—glassy carbon electrode/multi-walled carbon nanotubes-horseradish peroxidase; **GCE/MWCNT/CuPc**—glassy carbon electrode/multi-walled carbon nanotubes/copper phthalocyanine; **HMDE**—hanging dropping mercury electrode; **HRP/PDMA-PSS/Au**—horseradish peroxidase/poly(2,5-dimethoxyaniline)-poly(4- styrene sulfonic acid)/Au; **HF-PGE/CuO/MWCNTs–IL**—hollow fiber-pencil graphite electrode/copper oxide/multi-walled carbon nanotube-ionic liquid; **LOD**—limit of detection; **LSV**—linear sweep voltammetry; **MIP/GNPs-PGE**—molecularly imprinted polymer/gold nanoparticles-pencil graphite electrode; **MIP-MOF**—molecularly imprinted polymer-metal organic framework; **MIPPy/Au**—molecularly imprinted polypyrrole/Au; **NiAl-LDH/Pt**—NiAl-layered double hydroxide/Pt; **PBS**-phosphate buffer solution; **PPY-MIP/Au**—polypyrrole-molecularly imprinted polymer/Au; **PPy-MIP/ZnO**—polypyrrole-molecularly imprinted polymer/zinc oxide; **SCE**—saturated calomel electrode; **SPE**—screen printed electrode; **SPE/Chi/CNO/TYR**—screen printed electrode/chitosan/carbon nano-onions/tyrosinase; **SWV**—square wave voltammetry.

**Table 2:** An overview of electrochemical techniques, performance characters, medium of measurement and sample matrix for the determination of lindane.

Electrode	Technique	Medium	Potential	LOD	Linear Range	Matrix	Reference
PANI-microbial biosensor	Amperometry		0.40 V	6.90 nM	0.02–1.72 µM		[29]
α-MnO_2_-NW/GCE	Amperometry/DPV	0.05 M TBAB solution in 60:40 methanol–water	−1.45 V vs. Ag/AgCl	114 nM	1.10–510 µM	Spiked tap water	[75]
Vitreous carbon	CV, SWV	0.1 M of TBAB in ethanol	−2.0 V vs. Ag/AgCl	50.0 nM			[73]
CA/GCE	CV, DPV	0.05 M TBAB 60:40 methanol–water	−1.50 V vs. Ag/AgCl	37.0 µM	50.0–1000 µM	Lindane lotion	[64]
Silver	CV	ACN, DMF, EtOH, ACN–H_2_O, DMF–H_2_O, EtOH–H_2_O 0.050 M TBABF_4_	−0.89 V–−1.65 V vs. SCE				[67]
CuO–MnO_2_	DPV	0.05 M TBAB solution in 60:40 methanol–water	−1.50 V vs. Ag/AgCl	4.80 nM	1.0−700 µM	Tap water	[32]
NiCo_2_O_4_/GCE	DPV	0.05 M TBAB solution in 60:40 (v/v) methanol–water	−1.50 V vs. Ag/AgCl	5.90 µM	10.0–170 µM	Tap water	[77]
Streptomyces strain M7 biosensor	EIS			0.03 µM			[30]
MWCNT-MIP-Cu	Potentiometry			0.10 nM	1.0 nM–1.0 mM	water, fruits and vegetables	[27]
GCE/PANI-ZnO, GCE/PANI-Fe_3_O_4_, GCE/Nylon 6,6/MWCNT/ZnO, GCE/Nylon 6,6/MWCNT/^Fe3O4^ Concentration	SWV	60:40 methanol/water containing 0.05M TBAB	−0.80 V vs. Ag/AgCl	32.0 nM	9.90 pM–5.0 µM	Tap waters	[76]
vitreous carbon	SWV	0.10 M Bu_4_NBF_4_ in DMF ((DPA as mediator)	−1.73 V vs. Ag/AgCl		40.0–1000 µM		[70]
GCE		0.10 M TBABF_4_ in DMF	−1.40 V vs. Ag/AgCl				[71]
Hg/Pt		0.10 M TBAB in DMSO	−1.52 V vs. SCE			Sewage sludge, soil	[74]

Abbreviations: **ACN**—acetonitrile; **CA/GCE**-cellulose acetate/glassy carbon electrode; **CV**—cyclic voltammetry; **DMF**—n,n-dimethylformamide; **DMSO**—dimethyl sulfoxide; **DPA**—9,10-diphenylanthracene; **DPV**-differential pulse voltammetry; **EIS**—electrochemical impedance spectroscopy; **GCE**—glassy carbon electrode; **GCE/Nylon 6,6/MWCNT**—glassy carbon electrode/nylon 6,6/multi-walled carbon nanotubes; **GCE/PANI**—glassy carbon electrode/polyaniline; **LOD**—limit of detection; **MWCNT-MIP-**—multi-walled carbon nanotube-molecularly imprinted polymer; **PANI**—polyaniline; **SCE**—saturated calomel electrode; **SWV**—square wave voltammetry; **TBAB**—tetraethylammonium bromide; **α-MnO_2_-NW/GCE**—α-manganese oxide nanowire/glassy carbon electrode.

**Table 3:** An overview of different electroanalytical techniques, measurement conditions, analytical performance and electrode material used for the determination of bentazone.

Electrode	Technique	Medium	pH	Potential	LOD	Linear Range	Matrix	Reference
GCE	FIA/Amperometry	ABS	4.5	1.10 V vs. Ag/AgCl	1.0 µM	2.50–50.0 µM	estuarine water	[93]
MWCNT-IL/RGO/SiC/CILE	Continuous Coulometric FFT CV	0.05 M PBS	4.5	0.70 V	0.25 nM	1.0–150 nM		[88]
PANI-β-CD/fMWCNT	CV	PBS	6	0.85 V vs. Ag/AgCl	1.60 µM	10.0–80.0 µM	River water	[31]
PANI-CPE	CV	0.05 M PBS	6.9					[92]
BDD	DPV	B-R	4	1.07 V vs. Ag/AgCl	0.50 µM	2.0–100 µM	River water	[89]
GCE	DPV	0.20 M ABS	3.4	0.94 V vs. Ag/AgCl	10.0 µM	15.10–2.30 µM	Basagran	[86]
β-CD-GCE	DPV	0.10 M BR	6	0.93 V vs. Ag/AgCl		2.0–14.0 mM		[91]
poly-n-AcMnODEAETPc-GCE	SWV	0.10 M PBS	5	0.80 V vs. Ag/AgCl	0.25 µM	50.0–750 µM		[87]
SPE	SWV	0.10 M PBS	7	0.71 V	34.0 nM	0.19–50.0 µM	Ground and lake water	[90]

Abbreviations: **ABS**—acetate buffer solution; **BDD**—boron doped diamond; **B-R**—Britton-Robinson; **CV**—cyclic voltammetry; **DPV**—differential pulse voltammetry; **FIA**—flow injection analysis; **GCE**—glassy carbon electrode; **LOD**—limit of detection; **MWCNT-IL/RGO/SiC/CILE**—multi-walled carbon nanotube-ionic liquid/reduced graphene/silicone carbide/carbon ionic liquid electrode; **PBS**—phosphate buffer solution; **PANI-β-CD/fMWCNT**—polyaniline-β-cyclodextrine/functionalized multi-walled carbon nanotube; **PANI-CPE**—polyaniline-carbon paste electrode; **poly-n-AcMnODEAETPc-GCE**—poly-n-manganese acetate octakis-(2-diethyaminoethanethiol)phthalocyanine-glassy carbon electrode; **SPE**—screen printed electrode; **SWV**—square wave voltammetry; **β-CD-GCE**—β-cyclodextrine-glassy carbon electrode.
